# Third Ventricular Chordoid Glioma With TTF‐1 Positivity: A Diagnostic and Therapeutic Challenge

**DOI:** 10.1002/ccr3.73436

**Published:** 2026-09-06

**Authors:** Zhiwei Shen, Buyi Zhang, Xiangdong Zhu

**Affiliations:** ^1^ Department of Neurosurgery, Second Affiliated Hospital, School of Medicine Zhejiang University, Clinical Research Center for Neurological Diseases of Zhejiang Province Hangzhou China; ^2^ Department of Histopathology, Second Affiliated Hospital, School of Medicine Zhejiang University Hangzhou China

**Keywords:** chordoid glioma, craniopharyngioma, pituicytoma, third ventricle

## Abstract

Third ventricular chordoid glioma is a rare tumor often misdiagnosed due to nonspecific imaging. TTF‐1 immunoreactivity is critical for definitive diagnosis. Management should be individualized, balancing gross resection, adjuvant radiotherapy, and surveillance depending on surgical risk and recurrence potential.

## 1 Case Presentation

**FIGURE 1:**
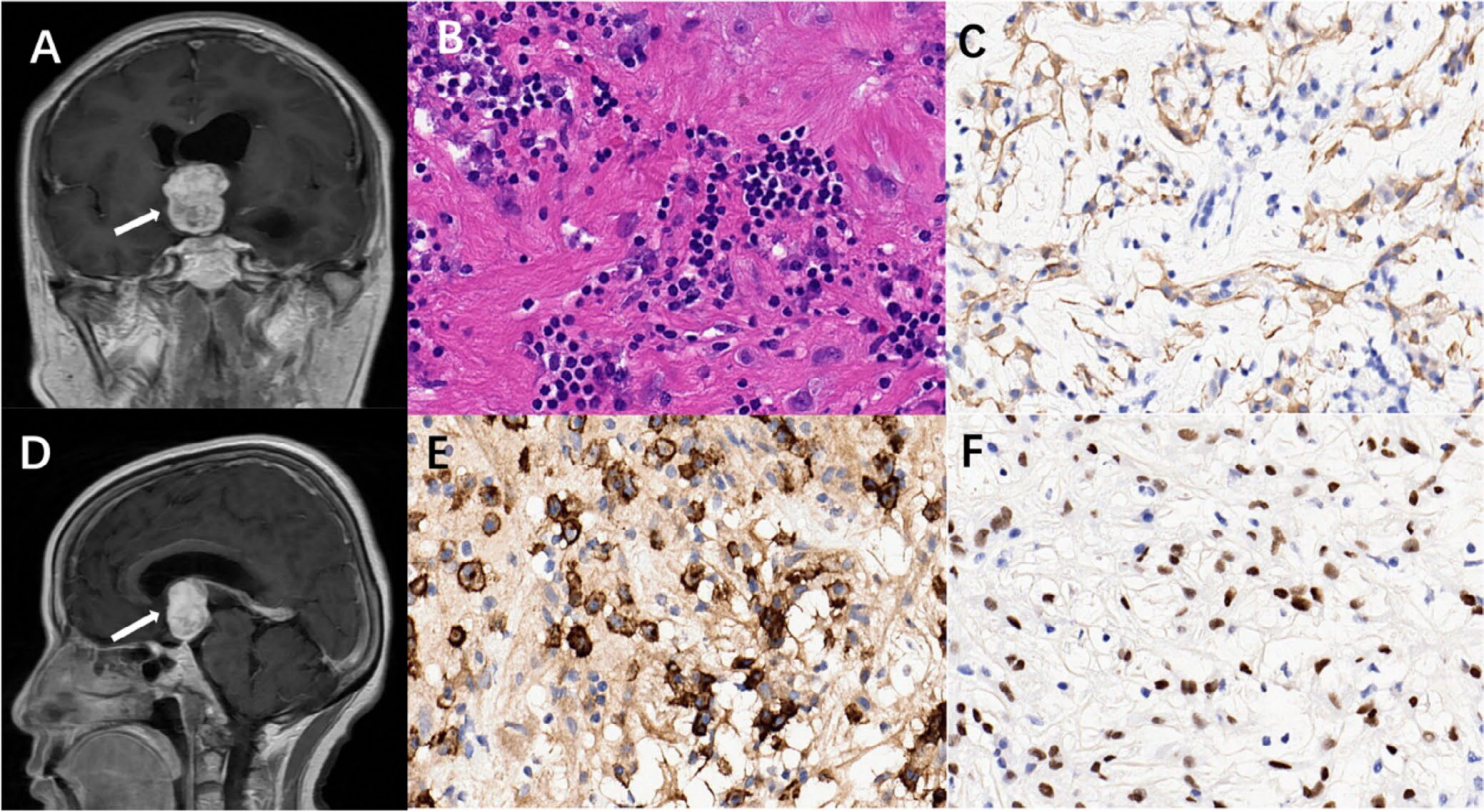
Magnetic resonance images of the head showing a heterogeneously enhancing mass in the suprasellar region (A, coronal view) and the third ventricle (D, sagittal view); hematoxylin–eosin staining showing clusters or cords of epithelioid tumor cells surrounded by variable mucinous stroma (B). Immunohistochemical staining showing that the tumor cells stained positive for GFAP (C), EMA (E), TTF‐1 (F) (magnification × 400, all panels).

A 59‐year‐old woman presented with a 5‐day headache, vomiting, and bradyphrenia. Neurological examination revealed impaired orientation and memory. Visual function and endocrine status were normal. She had a 2‐year history of Type 2 diabetes. CT showed a soft‐tissue mass in the suprasellar region and third ventricle without calcification. Contrast‐enhanced MRI demonstrated a 29 × 23 × 31 mm heterogeneously enhancing mass with clear borders and perilesional edema (Figure 1A,D). Blood and endocrine tests were unremarkable. The main differential diagnoses include craniopharyngioma and pituicytoma. Craniopharyngiomas derive from hypophyseal duct remnants and commonly present with calcification and T1WI hyperintensity. Pituicytomas show homogeneous enhancement and lack a posterior pituitary bright spot on T1WI. Calcification rarely occurs in both pituicytoma and chordoid glioma and cannot differentiate them. Gross total resection was performed via transcallosal approach with neuro‐endoscopic assistance. The tumor was hypervascular and appeared adherent to adjacent structures. Histopathology showed cords of epithelioid cells in mucinous stroma with dense lymphocytic infiltration (Figure 1B). IHC was positive for glial fibrillary acidic protein (GFAP) (Figure 1C), epithelial membrane antigen (EMA) (Figure 1E), TTF‐1 (Figure 1F) (magnification×400, all panels). Ki‐67 was 2%. The patient recovered uneventfully and was discharged on postoperative day 7. MRI on the 3rd postoperative day confirmed complete tumor resection. She received adjuvant radiotherapy with IMRT PTV DT 56Gy/26F according to the clinical experience of chordoid glioma management [1]. At 1‐year follow‐up, neurological function fully recovered, and imaging remained stable without recurrence.

## 2 Discussion

Chordoid glioma is a rare circumscribed astrocytic glioma with Grade II malignancy [2]. Magnetic resonance imaging (MRI) typically shows a well‐defined enhancing mass that is hypointense to isointense on T1‐weighted images and isointense to hyperintense on T2‐weighted images with marked homogeneous enhancement [3]. Imaging clues, including absent calcification, help distinguish this tumor from craniopharyngioma.

Histopathological evaluation is necessary for a definitive diagnosis of chordoid glioma. TTF‐1 immunoreactivity is a highly specific and reliable marker for the definitive diagnosis of chordoid glioma and helps distinguish it from craniopharyngioma. Furthermore, the diffuse positivity for glial fibrillary acidic protein, epithelial membrane antigen, and CD34 confirmed the diagnosis of chordoid glioma; these markers are typically negative in pituicytoma. Even with complete gross total resection, chordoid glioma carries documented low but measurable local recurrence risk [1]. Currently, no consensus exists on adjuvant radiotherapy following gross total resection for chordoid glioma, and active surveillance alone serves as a standard alternative. The deep third ventricular location renders repeat salvage surgery extremely high‐risk. We weighed benefits and risks: 56Gy IMRT can reduce recurrence risk, yet it may cause complications such as hypothalamic injury, radiation necrosis, and neurocognitive decline. This radiotherapy decision was a balanced clinical judgment. In any case, gross total resection and long‐term follow‐up are essential.

In summary, third ventricular chordoid glioma is a rare, diagnostically challenging tumor. TTF‐1 is a reliable diagnostic marker. Treatment selection after surgery requires individualized judgment.

## Author Contributions

**Zhiwei Shen:** writing – original draft. **Buyi Zhang:** methodology, software. **Xiangdong Zhu:** resources, supervision.

## Funding

The authors have nothing to report.

## Consent

A written informed consent was obtained from the patient to publish this report in accordance with the journal's patient consent policy and is available upon request.

## Conflicts of Interest

The authors declare no conflicts of interest.

## Data Availability

Data available on request from the authors.
